# A Design of a Time Synchronization Protocol Based on Dynamic Route and Forwarding Certification

**DOI:** 10.3390/s20185061

**Published:** 2020-09-06

**Authors:** Dejing Zhang, Yuan Yuan, Yanqing Bi

**Affiliations:** School of Mechanical, Electrical & Information Engineering, Shandong University, Weihai 264209, China; 201736334@mail.sdu.edu.cn (Y.Y.); yanqingbi@mail.sdu.edu.cn (Y.B.)

**Keywords:** wireless sensor networks, time synchronization protocol, dynamic routing, forwarding certification

## Abstract

Time synchronization is a key technique in large-scale wireless sensor network applications. In order to tackle the problems of multi-hop synchronization error accumulation, clock frequency skew swinging, and network topology changes, a time synchronization protocol based on dynamic routing and forwarding certification (DRFC-TSP) is proposed in this paper. During the time synchronization process, a reference node with fewer synchronization hops and a more stable clock frequency is selected for every single hop, in order to obtain the best synchronization route. In this way, synchronization error accumulation can be restrained and the impact of clock frequency skew swinging on the time synchronization precision can be reduced. Furthermore, changes of the network topology can be well adapted by dynamic routing, in which the reference node is updated in every synchronization round. In the forwarding certification process, the status of nodes forwarding synchronous information outwards is authored by information exchange between neighboring nodes. Only synchronous information of the certificated nodes with a better performance can be forwarded. The network traffic can be decreased and the time synchronization precision can also be ensured, even with less energy consumption. Feasibility testing in large-scale wireless sensor networks is verified on NS2 simulation and more performances are evaluated on an embedded Linux platform.

## 1. Introduction

Time synchronization is a fundamental technology in wireless sensor networks (WSN) [1,2]. Due to the collaboration features [3] of WSN nodes, it is important for each node to synchronize accurately [4]. Considering the particular demands [5,6,7] compared with the internet in terms of energy consumption limits and synchronization precision, traditional time synchronization protocols are not suitable for WSN. A variety of typical protocols have been proposed since the time synchronization subject of WSN was defined, such as the timing-sync protocol for sensor networks (TPSN) [8], delay measurement time synchronization (DMTS) [9], flooding time synchronization protocol (FTSP) [10], reference broadcast synchronization (RBS) [11], and pairwise broadcast synchronization (PBS) [12]. These protocols directly synchronize nodes’ clocks or trigger the process according to reference clock time information.

These typical protocols can be perfectly applied in a one-hop situation, but they cannot satisfy the synchronization needs in large-scale WSN. Meanwhile, the clock frequency skew swinging [13] that occurs in the long-running is another problem that is currently ignored. Accumulation error will increase severely if repeating one hop synchronization process between neighboring nodes in the network.

Therefore, based on typical protocols, several time synchronization protocols for large-scale WSN were put forward. For example, the dynamic route list based timing-sync protocol (DRL-TSP) [14] is a strategy that chooses a parent node in the last hop as the synchronization reference for each node, which provides the minimal hops from the root node by dividing the network into a hierarchical structure. Efficient and simple algorithm for time synchronization (E-SATS) [15] is proposed for reducing computations and energy consumption. However, for the WSN with a limited energy, a fixed hierarchical structure or a settled clustered structure results in a strong dependency on topology, which will consume extra energy in the process of building and fixing the established structure. Furthermore, it cannot react rapidly if the topology changes [16,17,18].

On the basis of the current time synchronization research, in order to tackle the problem of multi-hop error accumulation, the clock frequency skew influence, and the network topology changing, a time synchronization protocol named the time synchronization protocol based on dynamic routing and forwarding certification (DRFC-TSP) for large-scale WSN is proposed. In this protocol, by acquiring optimal reference time information in each hop instead of employing a fixed pre-established structure, every node can obtain an optimum route from the root node, which will ensure that the synchronization precision is good and allow adaptations to topology changes. The certification of time information forwarding is evaluated by a forwarding certification strategy. Therefore, the bad performance nodes do not forward their packets, and network traffic and energy consumption can be decreased.

The rest of the paper is organized as follows: Section 2 gives an introduction to the typical time synchronization models in large-scale WSN; Section 3 describes details on the algorithm of DRFC-TSP; Section 4 provides the design principles of realization on an embedded Linux platform; Section 5 illustrates the results of the simulation and field experiment; and the final section presents the conclusion.

## 2. Analysis of Time Synchronization Models in Large-Scale WSN

According to the time synchronization information exchanging methods, there is a hierarchical model and clustered model [19,20,21] in large-scale WSN. The hierarchical model is appropriate for protocols such as TPSN, in which information exchange occurs in pairs. In this model, the network is divided into a one-hop level structure, and nodes in one level synchronize with nodes in the next level. The clustered model is suitable for protocols such as FTSP, in which nodes obtain the synchronization information broadcasted by the reference nodes. In this model, a cluster structure is established at first and a head node is selected as the time reference in each cluster. Then, the head node broadcasts the time synchronization information to each node by multi-hop forwarding.

**Figure 1:**
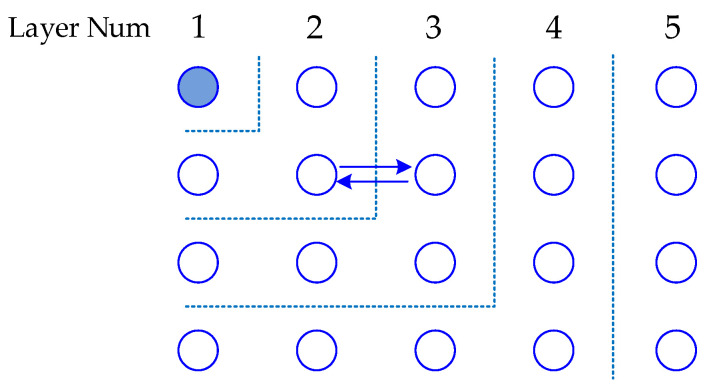
Hierarchical model.

A hierarchical model is shown in Figure 1. The first phase involves creating a hierarchy structure, and time synchronization is then carried out between levels. The root node launches a synchronization process and the information is delivered level by level. In this way, every node synchronizes with its parent node from the upper level by a one-hop process. In the level established phase, the level number should be the minimum, in order to reduce multi-hop error accumulation. This strategy will result in much redundant information exchange and aggravate the network traffic. Meanwhile, it strongly depends on the topology.

**Figure 2:**
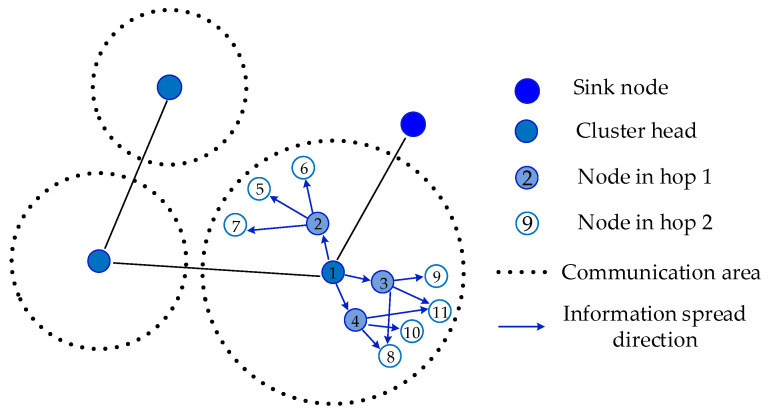
Clustered model.

A clustered model is shown in Figure 2. The synchronization is accomplished by cluster head and intracluster synchronization. After cluster heads synchronize, each head becomes a local root node and sends time information in its own clusters, where the synchronized nodes forward synchronization information to every node hop by hop. In the forwarding process, synchronization information is received by all of the neighbors. However, most of the information is redundant. This strategy is more adaptable to topology changes, but causes severe energy waste.

## 3. Design of a Time Synchronization Protocol Based on Dynamic Routing and Forwarding Certification

**Figure 3:**
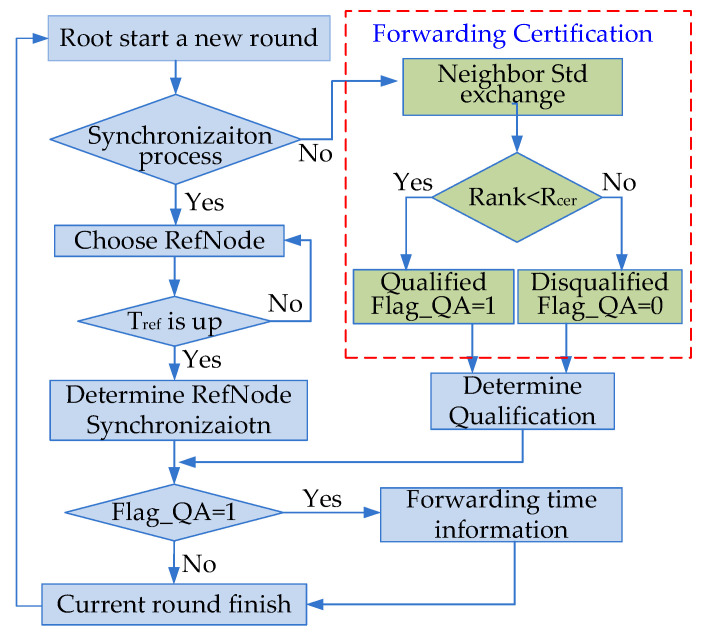
Flow chart of the time synchronization protocol based on dynamic routing and forwarding certification (DRFC-TSP).

The proposed time synchronization protocol DRFC-TSP is composed of two processes: the time synchronization process during which the node modifies its clock according to the reference information, and the forwarding certification process during which certain nodes are authorized to conduct forward certification. Two process phases are initiated by the root node, and the other nodes accomplish the corresponding process. The realization of DRFC-TSP is shown in Figure 3.

### 3.1. Time Synchronization Based on Dynamic Routing

In the time synchronization process, the root node initiates a new round of synchronization as the reference time. The others will accomplish information receiving; synchronization round recording; and reference synchronization information selecting, time synchronizing, and performance information caching. After accomplishing these processes, the node with forwarding certification should continue to forward time synchronization messages.

A time variable $T_{ref}$ is set for choosing the reference node. During the time of $T_{ref}$, a node without synchronization continually receives information from reference candidates. When $T_{ref}$ is increased, the candidate node with the best performance will be selected as the reference. By choosing the best reference in each hop, an optimum synchronization route will be ensured. The parameter called *Std* is defined as a measurement of the node performance, which is related to the hops to root node, clock frequency performance, and value of *Std* in the last synchronization round. According to *Std*, the best candidate will be selected as the reference. *Std* is defined as follows:

(1) $${Std}^{i}=\lambda \left(H\right)\cdot \mu \left(F\right)\cdot \eta \left({Std}^{i-1}\right)$$

where ${Std}^{i}$ is the performance parameter in round *i*, λ(H) is a variable determined by the hop number *H*, μ(F) is a variable determined by the frequency skew F in the current synchronization round, and $\eta \left({Std}^{i-1}\right)$ is the performance parameter in round *i*−1. A node with a good performance should have a smaller number of hops and a more stable clock frequency. Therefore, the node with a better node clock performance has a smaller *Std* parameter. Additionally, in order to achieve proper synchronization performances in different practical applications, *Std* should be adjusted according to the influence of hops and frequency skew in the specific network environment.

### 3.2. Authorization of Node Forwarding Certification

In the process of dynamic routing for time synchronization, nodes to be synchronized always select the best performance nodes as their synchronization references. Therefore, nodes with a poor performance have no chance of being selected as the references, even though the neighbors receive information from these poor nodes. In such a condition, network traffic will be heavily burdened due to large amounts of redundant information and energy is wasted. In order to improve the performance, the authorization of node forwarding certification is put forward.

A threshold marked $R_{cer}$ is set as the evaluation index, which represents the percentage of qualified nodes in the neighbor domain that are eligible to be put forward. $R_{cer}$ can be set according to the status of energy consumption. Each node compares its *Std* with the others’ and ranks itself in the neighbor domain. When its rank is larger than $R_{cer}$ in the neighbor domain, its forwarding certification will be terminated. Due to this strategy, useless information transmission will be effectively controlled and the energy consumption will be reduced.

Flag_QA is defined as the mark of forwarding certification. In the first round of time synchronization, all nodes are qualified to forward information, that is, the Flag_QA of each node is set to 1. After several rounds, the performance parameters of each node in previous rounds have been cached. Then, the process of forwarding certification is initiated. Each node broadcasts its *Std* and receives others to rank itself in the neighbor domain. If its rank is less than $R_{cer}$, Flag_QA is set to 1, which means that this node needs to forward its time synchronization information after completing time synchronization; otherwise, Flag_QA is set to 0, which means that this node just ends this round after synchronization, without forwarding time synchronization information to others.

### 3.3. Solution for the Reference Node Void Problem

Since the parts of nodes with a poor performance are disqualified from forwarding synchronization information, there is probably a problem that some nodes lack synchronization references; that is, all of the previous hop nodes within the communication range of a certain node are disqualified from forwarding time synchronization information, so that the node cannot synchronize because time synchronization information is not received in this round.

To tackle the reference node void problem, $T_{wait}$ is defined as the maximum waiting time. During $T_{wait}$, the nodes to be synchronized passively wait for time synchronization information. If there is no information received after $T_{wait}$, the node is considered to have the status of a reference node void. In this case, the node will initiate a synchronous request to its neighbors. Then, all nodes that have received this request will reply. The node will be synchronized with the best performance reference node.

## 4. Realization of DRFC-TSP on an Embedded Linux Platform

### 4.1. Development and Testing Environment

The protocol program is developed and compiled on Ubuntu 12.04, and then implemented in the embedded system with Linux Kernel 2.6. Nodes communicate via the Wi-Fi wireless card with the IEEE 802.11 g communication protocol, which works in a 2.4 GHz public frequency band and reaches the maximum transfer rate of 20 Mbit/s or more. An ad-hoc network with the Smart210 Linux embedded platform is integrated, which communicates by Wi-Fi, the ad hoc on-demand distance vector routing (AODV) [22] WSN routing protocol, and the IP routing protocol embedded in the Linux operating system [23]. The User Datagram Protocol (UDP) communication protocol is adopted. In socket network programming, the time information in the synchronization process is acquired by calling relevant time system functions. Specifically, the data structure Timeval of the Linux kernel is used, and two functions, consisting of gettimeofday() and settimeofday(), are called to obtain and modify the system time.

### 4.2. Realization Process

#### 4.2.1. Synchronization Process of the Root Node

The clock of the root node is considered to be the reference time; that is, the time offset is always 0 and the frequency skew is always 1. The root node will provide a time reference to the network and initiate the time synchronization process. The forwarding certification round will be carried out after *N* rounds of time synchronization.

During a synchronization round, only the root node can update the round number. When a new round of time synchronization or forwarding certification is carried out, the synchronization round number is increased by 1.

When a new round of synchronization is initiated, the current round number should be check. If the round number is the integral multiple of N, forwarding certification should be initiated in the current round; otherwise, the time synchronization process is carried out.

#### 4.2.2. Synchronization Process of the Non-Root Node

**Figure 4:**
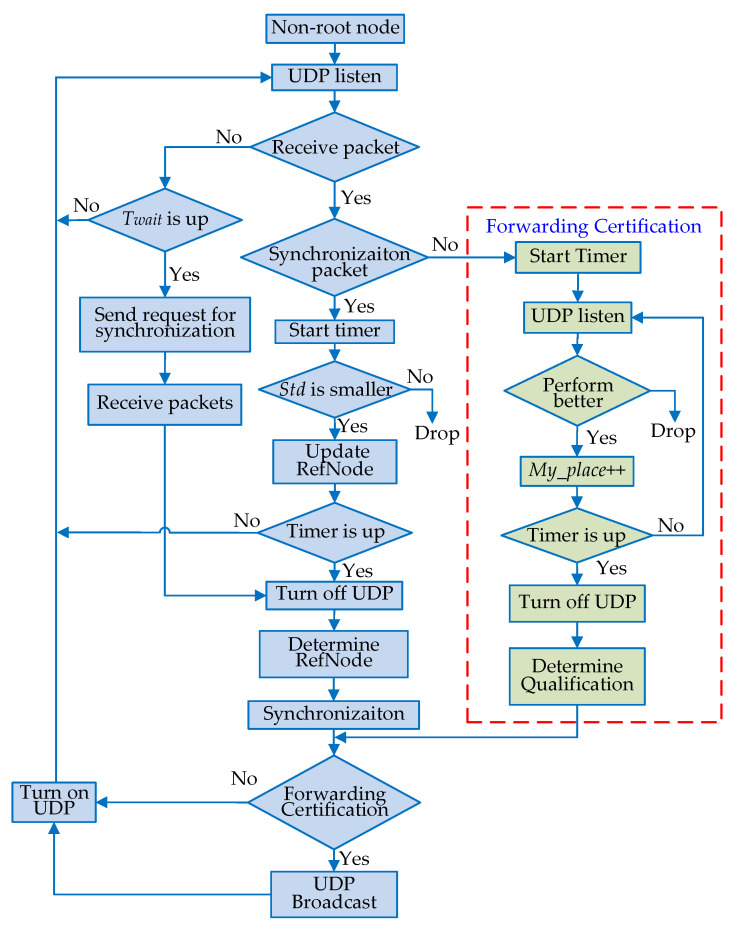
Synchronization flow chart of the non-root node.

The synchronization flow chart of the non-root node is shown in Figure 4.

(a) All of the non-root nodes keep the UDP server online to monitor the synchronization packet.
(b) When the node detects a packet, go to step (*d*); otherwise, go to step (*c*).
(c) If the timer for $T_{wait}$ is up, the node should send a request for synchronization to its neighbors because a void appears. After the packets from neighbors are received, go to step (*h*); otherwise, go to step (*a*).
(d) When the node to be synchronized detects a synchronization packet, it should determine whether the current message is a time synchronization packet or forwarding certification. If a time synchronization packet is received, then go to step (*e*); otherwise, it is a forwarding certification packet, so go to step (*k*).
(e) The timer of synchronization is turned on to count down $T_{ref}$.
(f) The node to be synchronized records several parameters in the received packet, such as the current round number, the hop count, *Std*, the sending time, etc. Furthermore, it needs to record the local time when the current packet is received. These parameters are tentatively set as the reference node information for the node. The node to be synchronized keeps on receiving other packets and compares synchronization information with its record. If the performance of the newly received synchronization message is better than the current record, the record about candidate reference nodes is updated; otherwise, the record remains unchanged.
(g) If the timer for $T_{ref}$ is up, go to step (*h*); otherwise, go to step (*a*).
(h) Turn off UDP monitoring. The node is synchronized according to the records and caching information in this round.
(i) If the node is forwarding certificated, go to step (*j*); otherwise, the node does nothing, so go step (*n*).
(j) Generate the time synchronization packet and forward it to the next hop. The current round of synchronization is finished, so go to step (*n*).
(k) The timer for certification is turned on.
(l) The number of neighbors is marked as My_Neighbor plus 1 when a node receives a certification packet. If the Std of the neighbor node is smaller than that of the current node, it is considered that the neighbor performs better, and the ranking parameter marked as My_Place of the current node is increased by 1; otherwise, it is considered that the neighbor performs worse, and My_Place remains unchanged.
(m) Determine whether the timer for certification has expired, if not, continue to step (*l*); otherwise, turn off UDP monitoring, and then compare the two parameters My_Place and My_Neighbor. If $\mathrm{My}\_\mathrm{place}\leq R_{\mathrm{cer}}\times \mathrm{My}\_\mathrm{neighbor}$, then the node’s performance is considered to be better than the threshold, so it is authorized with forwarding certification and set as Flag_QA = 1. If not, this node is not certificated and marked as Flag_QA with 0. Then, go to step (*i*).
(n) Restart the UDP monitor for packets of the next round, and then go to step (*a*).

## 5. Analysis of the Simulation and Test

Both a simulation and field experiment were carried out, in order to evaluate the performance of the proposed time synchronization protocol. The feasibility was tested on a simulation platform of NS 2.29. In addition, the time precision and topology adaptation of the proposed protocol were evaluated on the embedded Linux platform.

### 5.1. Simulation in NS2

#### 5.1.1. Analysis of the Reference Node Optimization

**Figure 5:**
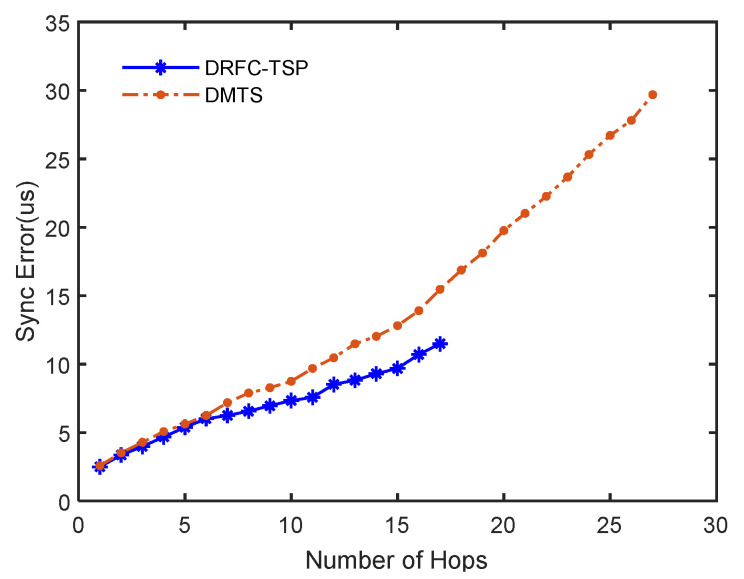
Performance of the accumulation error.

Parameter settings in the simulation are shown in Table 1. In this simulation, 300 nodes with a long enough $T_{ref}$ value (5 ms) were tested, which ensured that all reference candidates’ synchronization packets could be received. The performance of the accumulation error is shown in Figure 5. As can be seen, DRFC-TSP costed 17 hops for synchronizing the whole network, but DMTS without reference optimizing costed 27 hops. Moreover, the synchronization error of the edge node of DMTS was almost three times greater than that of DRFC-TSP. After six hops, the error of DMTS grew faster than that of DRFC-TSP. The simulation in NS2 shows that the hop number of a node achieving synchronization was smaller; that is, less energy was consumed in the time synchronization process in DRFC-TSP with reference node optimization. As is well known, the energy consumption of WSN was mainly in communication. Since DRFC-TSP can obtain more precise time by less hops than DMTS, several evaluations for the proposed protocol was performed.

#### 5.1.2. Influence of $T_{ref}$ on the Network Convergence Time

**Figure 6:**
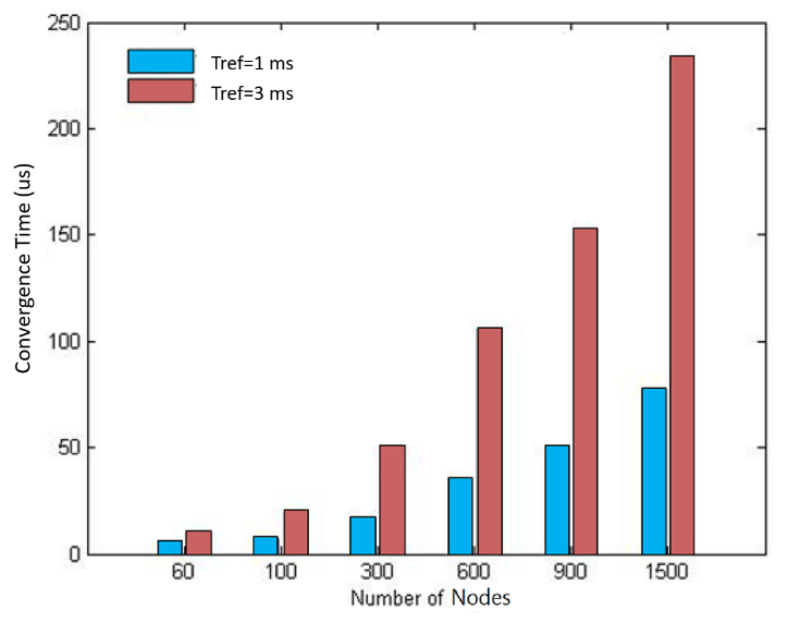
Convergence time of different numbers of nodes.

In the simulation, networks with 60, 100, 300, 600, 900, and 1500 nodes were tested separately, and the values of $T_{ref}$ were set to 1 and 3 ms, respectively. The simulation result is shown in Figure 6. As can be seen, the convergence time became longer when the number of nodes grew. In the simulation with the same number of nodes, the synchronization convergence time was positively related to $T_{ref}$. This shows that $T_{ref}$ was the main factor affecting the convergence time of the whole network.

#### 5.1.3. Influence of $T_{ref}$ on the Synchronization Precision

**Figure 7:**
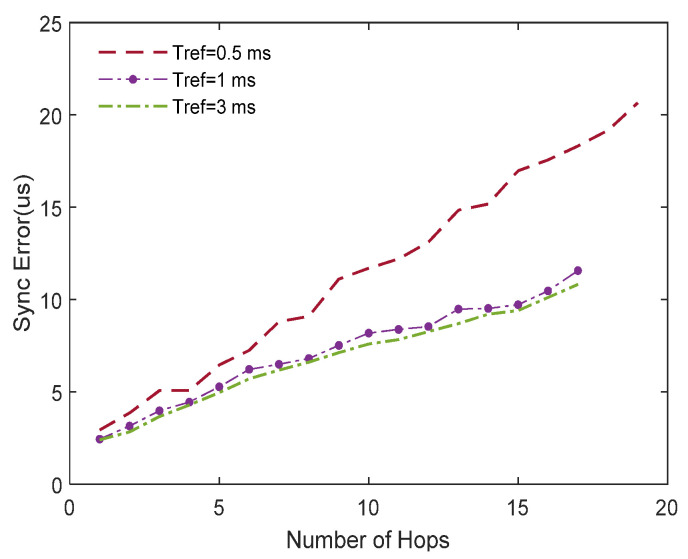
Synchronization error comparison of different *T_ref_.*

In the simulation, the network contained 300 nodes and the communication delay was a random variable with a mean of 1 ms and variance of 2 ms^2^. $T_{ref}$ was set as 0.5, 1, and 3 ms, respectively. The result is shown in Figure 7. In the simulation when $T_{ref}$ was 0.5 ms, the number of hops required to complete the time synchronization of the network was two times greater than when $T_{ref}$ was 1 or 3 ms. Meanwhile, the accumulation error of synchronization in the simulation where $T_{ref}$ was 0.5 ms was relatively larger than in the others and the growth was more obvious. Therefore, a larger $T_{ref}$ was beneficial for selecting a reference node with a better performance. However, the cumulative errors when $T_{ref}$ was 1 or 3 ms were nearly the same; that is, $T_{ref}$ was a factor of synchronization precision, but its influence became lower when $T_{ref}$ became bigger. In applications, an appropriate $T_{ref}$ should be selected to satisfy the need of the convergence time and synchronization precision.

### 5.2. Field Test on an Embedded Linux Platform

The field test used nine embedded Linux platforms as nodes, and carried out the porting of DRFC-TSP on the platform. The field tests on the multi-hop error accumulation, synchronization accuracy, and adaptability of the protocol to different topologies were evaluated. The frequency offset of the nine nodes relative to node 0 is shown in Table 2.

#### 5.2.1. Analysis of the Forwarding Certification Process

**Figure 8:**
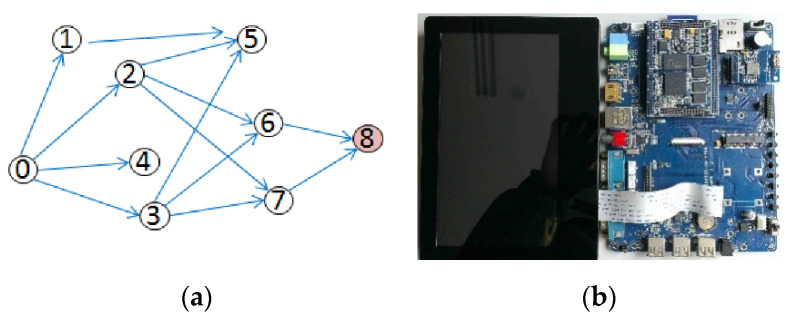
Distribution of nodes and the single node platform. (**a**) Distribution of nodes in the field test and (**b**) embedded Linux platforms as nodes in the test.

In the test, node 0 was set as the root node, and node 8 was the node to be synchronized. Nodes 1, 2, 3, and 4 were set as the second hop, being one hop from the root node, and nodes 5, 6, and 7 were set as the third hop, being two hops from the root node. The topology described above is shown in Figure 8.

When the time synchronization test finished, the parameters of each node were recorded and are shown in Table 3. This table shows that nodes 1, 4, and 5 were not certificated, and node 2 and 7 were selected as the reference nodes. Node 8 was synchronized with node 7. The test shows that the principle of forwarding certification in DRFC-TSP could introduce a better reference node for every hop. Therefore, node 8 had 3.03 ms time offset relative to node 0 in the test.

#### 5.2.2. Routing Influence on the Synchronization Precision

**Figure 9:**
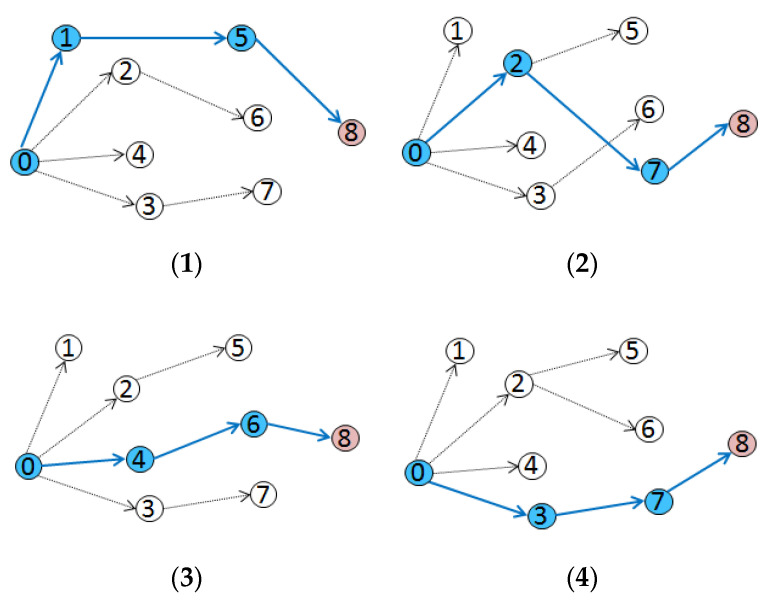
(**1–4**) Comparison of routes in multi-hop experiments.

In order to evaluate the influence of different routes on the synchronization precision, the four routes shown in Figure 9 were tested.

**Figure 10:**
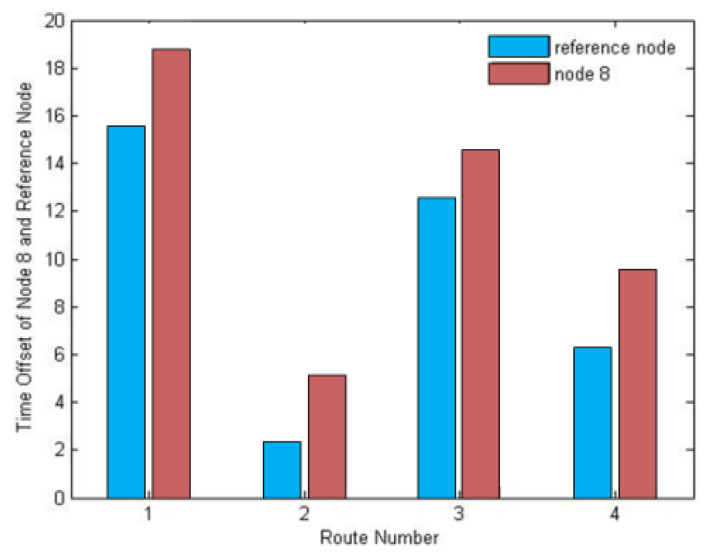
Time offset of node 8 and its reference of each route.

The routing established by DRFC-TSP in the tests is shown in Figure 9(2), and the others are designed routes. As can be seen from Figure 10, route 0-1-5-8 shown in Figure 9(1) has the worst performance of synchronization, while route 0-2-7-8 shown in Figure 9(2) is the best, and the other two routes 0-4-6-8 and 0-3-6-8 perform better than route 0-1-5-8. The result of these field tests can be predicted from Table 3. Due to the poor performance of nodes 1 and 5 in route 0-1-5-8, node 8 has the worst synchronization performance. In comparison, route 0-2-7-8 established by DRFC-TSP provides the best synchronization, since each node in this route optimizes its reference nodes.

#### 5.2.3. Adaptation of DRFC-TSP to the Different Topologies

**Figure 11:**
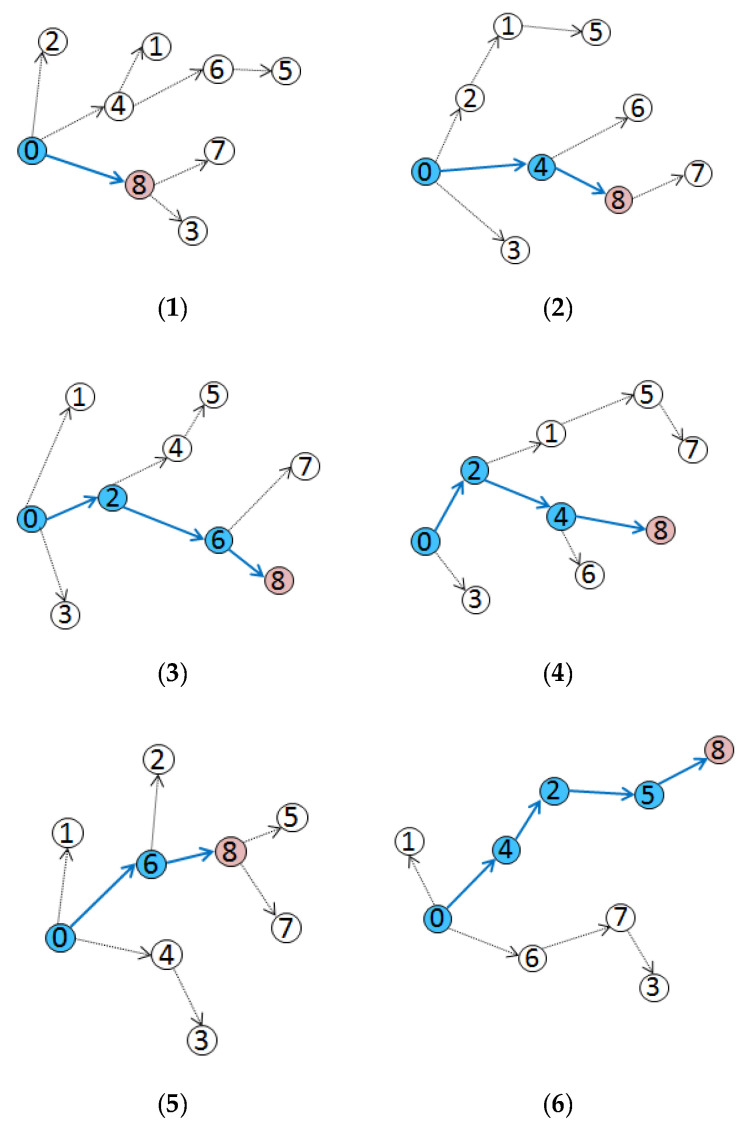
(**1–6**) Synchronization in six different topologies.

**Figure 12:**
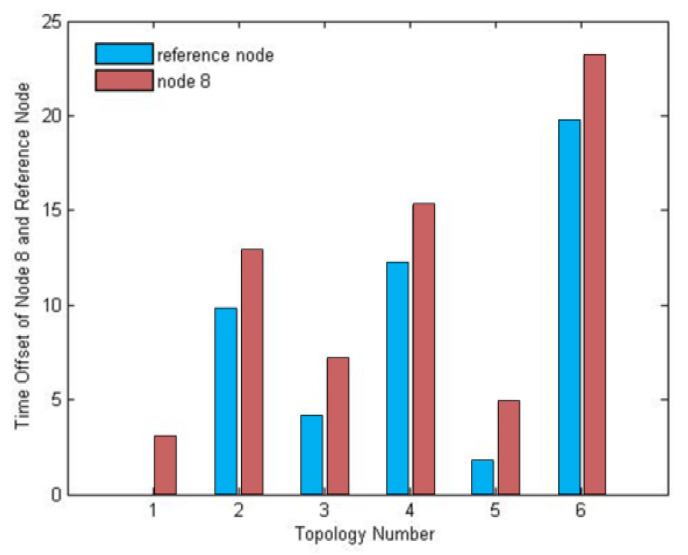
Comparison of synchronization in different topologies.

In order to evaluate the adaptation of DRFC-TSP to the different topologies, the six topologies shown in Figure 11 were simulated. The time offset of node 8 and its reference node is shown in Figure 12. In the topology shown in Figure 11(2) and topology shown in Figure 11(4), node 4 was selected as the reference node, although its offset was slightly larger compared with the cost of adding one hop. In the topology shown in Figure 11(5), node 8 selected node 6 as the reference node to synchronize, because the hop from root node 0 and time offset of node 6 were both the smallest. In the topology shown in Figure 11(3), nodes 2 and 6 exhibiting a small offset were selected as intermediate forwarding nodes, and this is the path with the least number of hops.

## 6. Conclusions

The proposed DRFC-TSP based on dynamic routing and node forwarding certification was designed to reduce accumulation error and the impact of clock frequency skew swinging on the time synchronization precision. Meanwhile, the reference node void coping mechanism can deal with synchronization process interruption to ensure that the synchronization process is reliable. The simulation and field test show that DRFC-TSP had good time synchronization performance. As shown, $T_{ref}\;\mathrm{and}\;R_{cer}$ in DRFC-TSP could be modified according to the requirement of precision and energy status. Moreover, the choice of functions for Std could affect the performance. How to strike optimal trades among these parameters in different applications, which is a direction for our future research. In addition, only several topologies were tested due to limited embedded Linux platforms. Although the proposed protocol could adapt them, further experiments in the large-scale environment are required in our future work.

## Figures and Tables

**Table 1:** Simulation parameters in NS2.

Parameter	Value
Node number	60/100/300/600/900/1500
Average distance of nodes	10 m
Wireless communication radius	20 m
Clock frequency	±50 ppm
Synchronization period	30 s
Simulation time	3000 s

**Table 2:** Frequency offset of embedded Linux platforms.

Num.	Wireless Card Mac Address	Frequency Offset Node 0 (ppm)
0 (Standard)	FC:4D:D4:6D:D7:F4	0
1	E0:2A:82:71:F4:2C	−115.5
2	02:25:65:38:34:F0	12.4
3	44:33:4C:49:41:DE	53.6
4	44:33:4C:45:37:BA	102.7
5	44:33:4C:49:50:BA	103.1
6	44:33:4C:49:42:0C	14.9
7	44:33:4C:49:41:EA	4.6
8	44:33:4C:46:D1:C9	82.8

**Table 3:** Parameters of embedded Linux platforms after the test.

Node Num.	Hops	Certificated or Not	Reference	Offset (ms)
1	1	No	0	8.24
2	1	Yes	0	2.34
3	1	Yes	0	6.24
4	1	No	0	9.79
5	2	No	2	7.55
6	2	Yes	2	1.69
7	2	Yes	2	0.26
8	3	-	7	3.03
